# Data on online and face-to-face course enrollments in a public research university during summer terms

**DOI:** 10.1016/j.dib.2020.105320

**Published:** 2020-02-22

**Authors:** Christian Fischer, Di Xu, Fernando Rodriguez, Kameryn Denaro, Mark Warschauer

**Affiliations:** a University of Tübingen, Germany; b University of California, Irvine, United States

**Keywords:** Higher education, Online courses, Distance education, Summer session, At-risk students

## Abstract

This data article includes information on institutional data at a large public research university in Southern California. In particular, data on undergraduate student enrollments in online and face-to-face courses during summer terms from 2014 to 2017 cumulating in 72,441 course enrollments from 23,610 undergraduate students in 433 courses is provided. This data includes additional information on the statistical models examining factors influencing student enrollment by course modality and the associations of course modality with course grades. This includes descriptive data and data derived from multi-level logistic regression analyses and multi-way fixed effects linear regression analyses. This data article is associated with the article “Effects of course modality in summer session: Enrollment patterns and student performance in face-to-face and online classes” [1].

**Table:** Specifications Table

Subject area	*Education*
More specific subject area	*Higher Education, Online Learning*
Type of data	*Tables*
How data was acquired	*Institutional data base of the University of California, Irvine*
Data format	*Analyzed, raw (upon request)*
Experimental factors	*Course enrollments and student grades by course modality*
Experimental features	*Logistic regression analysis, fixed effects modeling*
Data source location	*Irvine, CA, USA*
Data accessibility	*Data is within this article. Access to de-identified raw data is available upon request to the corresponding author for researchers with an approved IRB from their home institution.*
Related research article	*This article provides supplemental information for research published in the following study:* Fischer, C., Xu, D., Rodriguez, F., Denaro, K., & Warschauer, M. (2020). Effects of course modality in summer session: Enrollment patterns and student performance in face-to-face and online classes, *The Internet and Higher Education, 45*, 1-9

**Table:** 

**Value of the Data** • Insights on undergraduate course enrollments during summer terms may guide further development of summer course portfolios. • Insights on similarities and differences in student enrollment in online and face-to-face courses may guide adaptation of online course portfolios. • Details on the applied methodology and additional information on the applied statistical models may encourage replication studies and secondary data analyses.

## 1 Data

This Data in Brief article is associated with the article “Effects of course modality in summer session: Enrollment patterns and student performance in face-to-face and online classes” [1]. The data provided in this article sheds light on the demographics at a large research university during summer terms (Table 1, Table 2, Table 3, Table 8) and models associations of student- and course-level factors influencing course enrollments by course modality (Table 4, Table 5, Table 6, Table 7). In addition, the data describes associations of course modality with student course grades (Table 9, Table 10, Table 11, Table 12, Table 13, Table 14, Table 15, Table 16, Table 17, Table 18, Table 19).

**Table 1:** Descriptive information on all summer course enrollments.

	N	Mean/[%]	SD
Course grade	72,441	2.89	1.07
Online course	72,441	27.7%	
Female	72,152	56.3%	
White	69,556	12.0%	
Black	69,556	2.7%	
Asian	69,556	63.1%	
Hispanic	69,556	21.6%	
Native	69,556	0.5%	
English language learner	71,290	43.3%	
In-state student residency	72,438	76.4%	
Low-income status	71,290	33.6%	
First-generation status	71,290	51.4%	
Current college GPA	72,440	2.94	0.52
Years enrolled in college	72,434	2.22	1.39
Transfer student status	71,290	16.9%	
College admission score	61,532	225.72	28.80
Course repetitions	72,441	0.18	0.46
Number of online courses	72,007	0.85	1.25
Number students in course	72,441	92.37	66.90

**Table 2:** Descriptive course enrollment information for multi-level logistic regression analysis, full sample.

	All enrollments (N = 60,798)		Face-to-face enrollments (N = 43,855)		Online enrollments (N = 16,943)	
	Mean/[%]	SD	Mean/[%]	SD	Mean/[%]	SD
Online course	27.9%		0.0%		100.0%	
Female	58.0%		56.9%		61.0%	
White	10.0%		9.9%		10.4%	
Black	2.6%		2.5%		2.8%	
Asian	65.2%		64.7%		66.4%	
Hispanic	21.8%		22.5%		20.0%	
Native	0.4%		0.4%		0.4%	
English language learner	42.9%		41.8%		45.9%	
In-state student residency	76.2%		78.6%		70.0%	
Low-income status	35.2%		36.4%		32.2%	
First-generation status	51.7%		52.4%		50.0%	
Current college GPA	2.93	0.50	2.93	0.49	2.95	0.52
Years enrolled in college	2.25	1.02	2.26	1.01	2.22	1.04
Transfer student status	3.7%		3.9%		3.2%	
College admission score	225.52	28.79	224.80	29.30	227.38	27.34
Course repetitions	0.18	0.45	0.22	0.49	0.09	0.32
Number of online courses	0.93	1.30	0.86	1.21	1.12	1.48
Number students in course	95.08	68.22	84.07	66.00	123.59	65.55

**Table 3:** Descriptive course enrollment information for multi-level logistic regression analysis, restricted sample.

	All enrollments (N = 9741)		Face-to-face enrollments (N = 3436)		Online enrollments (N = 6305)	
	Mean/[%]	SD	Mean/[%]	SD	Mean/[%]	SD
Online course	64.7%		0.0%		100.0%	
Female	60.1%		60.0%		60.1%	
White	9.8%		9.5%		10.1%	
Black	2.7%		2.7%		2.7%	
Asian	65.9%		61.3%		68.4%	
Hispanic	21.1%		26.0%		18.5%	
Native	0.4%		0.6%		0.3%	
English language learner	44.6%		39.4%		47.4%	
In-state student residency	72.0%		80.1%		67.6%	
Low-income status	34.1%		39.6%		31.2%	
First-generation status	50.9%		54.8%		48.8%	
Current college GPA	2.92	0.53	2.88	0.54	2.95	0.52
Years enrolled in college	1.94	0.99	1.78	0.94	2.03	1.00
Transfer student status	2.1%		1.7%		2.4%	
College admission score	228.41	25.45	226.70	24.80	229.34	25.76
Course repetitions	0.11	0.35	0.14	0.37	0.10	0.33
Number of online courses	0.89	1.31	0.63	1.01	1.03	1.43
Number students in course	140.74	80.80	125.43	91.28	149.09	73.14

**Table 4:** Full sample multi-level logistic regression analysis in log-odds units, *N*_*Enrollments*_ = 60,798, *N*_*Courses*_ = 431.

	β	S.E.	z	p	95% CI	
Intercept	−12.2689	0.8841	−13.88	<0.001	−14.0017	−10.5361
Level 1						
Female (vs. Male)	0.2157	0.0400	5.39	<0.001	0.1373	0.2942
Race/Ethnicity (vs. White)						
Black	0.2003	0.1299	1.54	0.123	−0.0543	0.4549
Asian	−0.2439	0.0642	−3.80	<0.001	−0.3698	−0.1180
Hispanic	−0.1010	0.0745	−1.36	0.175	−0.2470	0.0450
Native	−0.2913	0.3076	−0.95	0.344	−0.8941	0.3116
English language learner	0.0393	0.0446	0.88	0.377	−0.0480	0.1267
In-state student residency	−0.3522	0.0569	−6.18	<0.001	−0.4638	−0.2406
Low-income status	−0.0382	0.0458	−0.83	0.404	−0.1279	0.0515
First-generation status	0.0717	0.0436	1.65	0.100	−0.0137	0.1571
Current college GPA	−0.0098	0.0407	−0.24	0.809	−0.0896	0.0699
Years enrolled in college	0.0640	0.0233	2.74	0.006	0.0183	0.1097
Transfer student status	0.4863	0.1293	3.76	<0.001	0.2329	0.7397
College admission score	0.3062	0.0239	12.79	<0.001	0.2592	0.3531
Course repetitions	0.3361	0.0563	5.97	<0.001	0.2258	0.4465
Number of online courses	0.2633	0.0177	14.86	<0.001	0.2285	0.2980
Level 2						
Number students in course	0.0138	0.0004	31.22	<0.001	0.0129	0.0146

**Table 5:** Full sample multi-level logistic regression analysis in odds ratio units, *N*_*Enrollments*_ = 60,798, *N*_*Courses*_ = 431.

	Odds ratio	S.E.	z	p	95% CI	
Intercept	0.0000	0.0000	−13.88	<0.001	0.0000	0.0000
Level 1						
Female (vs. Male)	1.2408	0.0497	5.39	<0.001	1.1471	1.3420
Race/Ethnicity (vs. White)						
Black	1.2218	0.1587	1.54	0.123	0.9471	1.5760
Asian	0.7836	0.0503	−3.80	<0.001	0.6909	0.8887
Hispanic	0.9039	0.0673	−1.36	0.175	0.7811	1.0460
Native	0.7473	0.2299	−0.95	0.344	0.4090	1.3656
English language learner	1.0401	0.0463	0.88	0.377	0.9531	1.1350
In-state student residency	0.7031	0.0400	−6.18	<0.001	0.6289	0.7861
Low-income status	0.9625	0.0440	−0.83	0.404	0.8800	1.0528
First-generation status	1.0743	0.0468	1.65	0.100	0.9864	1.1701
Current college GPA	0.9902	0.0403	−0.24	0.809	0.9143	1.0725
Years enrolled in college	1.0661	0.0249	2.74	0.006	1.0184	1.1160
Transfer student status	1.6262	0.2103	3.76	<0.001	1.2622	2.0952
College admission score	1.3582	0.0325	12.79	<0.001	1.2959	1.4235
Course repetitions	1.3995	0.0788	5.97	<0.001	1.2533	1.5628
Number of online courses	1.3012	0.0231	14.86	<0.001	1.2568	1.3472
Level 2						
Number students in course	1.0139	0.0004	31.22	<0.001	1.0130	1.0147

**Table 6:** Restricted sample multi-level logistic regression analysis in log-odds units, *N*_*Enrollments*_ = 9,741, *N*_*Courses*_ = 34.

	β	S.E.	z	p	95% CI	
Intercept	1.8922	0.3106	6.09	<0.001	1.2834	2.5011
Level 1						
Female (vs. Male)	0.2243	0.0526	4.26	<0.001	0.1211	0.3275
Race/Ethnicity (vs. White)						
Black	0.0388	0.1698	0.23	0.819	−0.2940	0.3716
Asian	−0.3399	0.0873	−3.90	<0.001	−0.5110	−0.1689
Hispanic	−0.3326	0.0993	−3.35	0.001	−0.5273	−0.1380
Native	−0.9827	0.3716	−2.64	0.008	−1.7110	−0.2544
English language learner	0.0337	0.0589	0.57	0.567	−0.0817	0.1490
In-state student residency	−0.1522	0.0753	−2.02	0.043	−0.2998	−0.0046
Low-income status	−0.0830	0.0603	−1.38	0.169	−0.2012	0.0353
First-generation status	0.0187	0.0577	0.32	0.746	−0.0944	0.1318
Current college GPA	0.0074	0.0522	0.14	0.887	−0.0949	0.1098
Years enrolled in college	0.0224	0.0312	0.72	0.473	−0.0387	0.0835
Transfer student status	0.2747	0.1812	1.52	0.130	−0.0805	0.6300
College admission score	0.1613	0.0334	4.82	<0.001	0.0957	0.2269
Course repetitions	0.7359	0.0825	8.92	<0.001	0.5742	0.8977
Number of online courses	0.2011	0.0238	8.44	<0.001	0.1544	0.2477
Level 2						
Number students in course	0.0115	0.0005	23.23	<0.001	0.0105	0.0125

**Table 7:** Restricted sample multi-level logistic regression analysis in odds ratio units, *N*_*Enrollments*_ = 9,741, *N*_*Courses*_ = 34.

	Odds ratio	S.E.	z	p	95% CI	
Intercept	6.6341	2.0608	6.09	<0.001	3.6089	12.1953
Level 1						
Female (vs. Male)	1.2514	0.0659	4.26	<0.001	1.1287	1.3874
Race/Ethnicity (vs. White)						
Black	1.0396	0.1765	0.23	0.819	0.7453	1.4501
Asian	0.7118	0.0621	−3.90	<0.001	0.5999	0.8446
Hispanic	0.7170	0.0712	−3.35	0.001	0.5902	0.8711
Native	0.3743	0.1391	−2.64	0.008	0.1807	0.7754
English language learner	1.0343	0.0609	0.57	0.567	0.9216	1.1607
In-state student residency	0.8588	0.0647	−2.02	0.043	0.7410	0.9954
Low-income status	0.9204	0.0555	−1.38	0.169	0.8177	1.0359
First-generation status	1.0189	0.0588	0.32	0.746	0.9099	1.1409
Current college GPA	1.0075	0.0526	0.14	0.887	0.9094	1.1161
Years enrolled in college	1.0227	0.0319	0.72	0.473	0.9620	1.0871
Transfer student status	1.3162	0.2386	1.52	0.130	0.9227	1.8776
College admission score	1.1750	0.0393	4.82	<0.001	1.1005	1.2546
Course repetitions	2.0875	0.1723	8.92	<0.001	1.7757	2.4540
Number of online courses	1.2227	0.0291	8.44	<0.001	1.1670	1.2811
Level 2						
Number students in course	1.0116	0.0005	23.23	<0.001	1.0106	1.0126

**Table 8:** Descriptive course enrollment information for multi-way fixed effects linear regression analysis.

	All enrollments		Face-to-face enrollments		Online enrollments	
	Mean/[%]	SD	Mean/[%]	SD	Mean/[%]	SD
Course enrollments of all students						
N	61,401		44,287		17,114	
Grade	2.90	1.07	2.87	1.06	2.97	1.08
Online course	27.9%		0.0%		100.0%	
Current college GPA	2.94	0.50	2.93	0.49	2.96	0.52
Years enrolled in college	2.24	1.02	2.26	1.01	2.21	1.03
Course repetitions	0.18	0.45	0.22	0.49	0.09	0.32
Number of online courses	0.93	1.29	0.85	1.21	1.12	1.47
Number students in course	95.45	68.43	84.43	66.24	123.95	65.70
Course enrollments of low-income students						
N	21,465		15,990		5475	
Grade	2.81	1.08	2.78	1.07	2.90	1.11
Online course	25.5%		0.0%		100.0%	
Current college GPA	2.84	0.47	2.83	0.46	2.85	0.48
Years enrolled in college	2.28	1.03	2.29	1.02	2.25	1.05
Course repetitions	0.21	0.49	0.25	0.53	0.11	0.34
Number of online courses	0.96	1.32	0.90	1.24	1.15	1.50
Number students in course	95.01	69.12	87.14	69.08	117.98	63.93
Course enrollments of first-generation college students						
N	31,596		23,084		8512	
Grade	2.82	1.08	2.79	1.07	2.91	1.11
Online course	26.9%		0.0%		100.0%	
Current college GPA	2.86	0.48	2.85	0.47	2.88	0.50
Years enrolled in college	2.27	1.00	2.28	0.99	2.26	1.03
Course repetitions	0.20	0.48	0.24	0.52	0.10	0.34
Number of online courses	0.97	1.32	0.90	1.24	1.19	1.50
Number students in course	95.29	68.74	85.87	67.89	120.84	64.41
Course enrollments of students in the lowest high school performance group						
N	14,105		10,596		3509	
Grade	2.70	1.11	2.68	1.11	2.77	1.13
Online course	24.9%		0.0%		100.0%	
Current college GPA	2.74	0.44	2.74	0.44	2.73	0.44
Years enrolled in college	2.76	1.11	2.76	1.09	2.74	1.17
Course repetitions	0.22	0.53	0.25	0.56	0.13	0.40
Number of online courses	1.13	1.40	1.04	1.30	1.43	1.64
Number students in course	88.74	65.42	80.10	63.61	114.82	63.90

**Table 9:** Two-way fixed effects linear regression analysis; student and course fixed effects; *N* = 61,401.

	Coef.	Robust S.E.	t	p	95% CI	
Intercept	−1.3967	0.1719	−8.13	<0.001	−1.7336	−1.0599
Online course	−0.1613	0.0332	−4.86	<0.001	−0.2263	−0.0963
Online course						
∗Low-income status	0.0330	0.0234	1.41	0.158	−0.0128	0.0789
Online course						
∗ First-gen status	0.0107	0.0226	0.48	0.634	−0.0335	0.0550
Online course						
∗ Medium HS performance (vs. Low HS performance)	−0.0656	0.0287	−2.29	0.022	−0.1218	−0.0094
Online course						
∗ High HS performance (vs. Low HS performance	−0.0868	0.0312	−2.79	0.005	−0.1479	−0.0258
Current college GPA	1.4198	0.0494	28.74	<0.001	1.3229	1.5166
Years enrolled in college	−0.0704	0.0116	−6.06	<0.001	−0.0932	−0.0476
Course repetitions	0.0281	0.0128	2.19	0.029	0.0029	0.0533
Number of online courses	−0.0008	0.0084	−0.10	0.924	−0.0174	0.0157
Number students in course	0.0001	0.0001	0.59	0.554	−0.0002	0.0003

**Table 10:** Three-way fixed effects linear regression analysis; student, course, and year fixed effects; *N* = 61,401.

	Coef.	Robust S.E.	t	p	95% CI	
Intercept	−1.3092	0.2824	−4.64	<0.001	−1.8628	−0.7556
Online course	−0.1681	0.0332	−5.07	<0.001	−0.2332	−0.1031
Online course						
∗Low-income status	0.0332	0.0234	1.42	0.155	−0.0126	0.0791
Online course						
∗ First-gen status	0.0117	0.0226	0.52	0.605	−0.0325	0.0559
Online course						
∗ Medium HS performance (vs. Low HS performance)	−0.0638	0.0286	−2.23	0.026	−0.1200	−0.0077
Online course						
∗ High HS performance (vs. Low HS performance	−0.0856	0.0311	−2.75	0.006	−0.1467	−0.0246
Current college GPA	1.4213	0.0493	28.83	<0.001	1.3246	1.5179
Years enrolled in college	−0.2185	0.3229	−0.68	0.498	−0.8514	0.4143
Course repetitions	0.0279	0.0128	2.18	0.029	0.0028	0.0531
Number of online courses	−0.0032	0.0086	−0.37	0.709	−0.0200	0.0136
Number students in course	0.0002	0.0001	1.37	0.172	−0.0001	0.0004

**Table 11:** Three-way fixed effects linear regression analysis; student, course, and instructor fixed effects; *N* = 61,401.

	Coef.	Robust S.E.	t	p	95% CI	
Intercept	−1.8425	0.2504	−7.36	<0.001	−2.3332	−1.3518
Online course	−0.0942	0.0520	−1.81	0.070	−0.1961	0.0076
Online course						
∗Low-income status	0.0282	0.0230	1.23	0.220	−0.0169	0.0733
Online course						
∗ First-gen status	0.0068	0.0221	0.31	0.757	−0.0365	0.0502
Online course						
∗ Medium HS performance (vs. Low HS performance)	−0.0552	0.0281	−1.97	0.049	−0.1102	−0.0002
Online course						
∗ High HS performance (vs. Low HS performance	−0.0813	0.0306	−2.66	0.008	−0.1413	−0.0214
Current college GPA	1.3993	0.0486	28.81	<0.001	1.3041	1.4945
Years enrolled in college	−0.0790	0.0122	−6.48	<0.001	−0.1029	−0.0551
Course repetitions	0.0275	0.0127	2.17	0.030	0.0027	0.0523
Number of online courses	0.0026	0.0083	0.31	0.754	−0.0137	0.0189
Number students in course	−0.0001	0.0002	−0.43	0.667	−0.0004	0.0003

**Table 12:** Four-way fixed effects linear regression analysis; student, course, instructor, and year fixed effects; full sample; *N* = 61,401.

	Coef.	Robust S.E.	t	p	95% CI	
Intercept	−2.3473	0.4246	−5.53	<0.001	−3.1795	−1.5151
Online course	−0.0962	0.0521	−1.85	0.065	−0.1983	0.0059
Online course						
∗Low-income status	0.0282	0.0230	1.23	0.219	−0.0168	0.0733
Online course						
∗ First-gen status	0.0071	0.0221	0.32	0.749	−0.0363	0.0505
Online course						
∗ Medium HS performance (vs. Low HS performance)	−0.0555	0.0281	−1.98	0.048	−0.1104	−0.0005
Online course						
∗ High HS performance (vs. Low HS performance	−0.0815	0.0306	−2.67	0.008	−0.1415	−0.0216
Current college GPA	1.3991	0.0485	28.84	<0.001	1.3040	1.4942
Years enrolled in college	0.6287	0.4878	1.29	0.197	−0.3273	1.5847
Course repetitions	0.0275	0.0127	2.17	0.030	0.0027	0.0523
Number of online courses	0.0000	0.0085	0.00	0.997	−0.0166	0.0167
Number students in course	0.0000	0.0002	−0.16	0.875	−0.0004	0.0003

**Table 13:** Four-way fixed effects linear regression analysis; student, course, instructor, and year fixed effects; low-income sample; *N* = 21,465.

	Coef.	Robust S.E.	t	p	95% CI	
Intercept	−1.4120	0.5880	−2.40	0.016	−2.5646	−0.2594
Online course	−0.1767	0.0764	−2.31	0.021	−0.3264	−0.0270
Current college GPA	1.3970	0.0741	18.85	<0.001	1.2518	1.5423
Years enrolled in college	−1.4270	0.5084	−2.81	0.005	−2.4237	−0.4304
Course repetitions	0.0803	0.0190	4.23	<0.001	0.0431	0.1175
Number of online courses	−0.0208	0.0136	−1.53	0.126	−0.0474	0.0059
Number students in course	−0.0002	0.0003	−0.75	0.452	−0.0008	0.0004

**Table 14:** Four-way fixed effects linear regression analysis; student, course, instructor, and year fixed effects; non-low-income sample; *N* = 39,936.

	Coef.	Robust S.E.	t	p	95% CI	
Intercept	−2.35969	0.42688	−5.53	<0.001	−3.1964	−1.5229
Online course	−0.12178	0.05495	−2.22	0.027	−0.2295	−0.0141
Current college GPA	1.39555	0.06269	22.26	<0.001	1.2727	1.5184
Years enrolled in college	0.66896	0.43523	1.54	0.124	−0.1842	1.5221
Course repetitions	0.00043	0.01690	0.03	0.980	−0.0327	0.0336
Number of online courses	0.01095	0.01074	1.02	0.308	−0.0101	0.0320
Number students in course	0.00002	0.00022	0.08	0.938	−0.0004	0.0005

**Table 15:** Four-way fixed effects linear regression analysis; student, course, instructor, and year fixed effects; first-generation student sample; *N* = 31,596.

	Coef.	Robust S.E.	t	p	95% CI	
Intercept	−3.4786	0.5097	−6.82	<0.001	−4.4778	−2.4795
Online course	−0.1671	0.0621	−2.69	0.007	−0.2887	−0.0454
Current college GPA	1.4720	0.0687	21.44	<0.001	1.3374	1.6066
Years enrolled in college	1.2429	0.4363	2.85	0.004	0.3878	2.0981
Course repetitions	0.0607	0.0160	3.79	0.000	0.0293	0.0921
Number of online courses	−0.0114	0.0120	−0.95	0.343	−0.0349	0.0121
Number students in course	0.0002	0.0003	0.83	0.409	−0.0003	0.0007

**Table 16:** Four-way fixed effects linear regression analysis; student, course, instructor, and year fixed effects; non-first-generation student sample; *N* = 29,805.

	Coef.	Robust S.E.	t	p	95% CI	
Intercept	−0.8502	0.3920	−2.17	0.030	−1.6186	−0.0818
Online course	−0.1131	0.0642	−1.76	0.078	−0.2389	0.0127
Current college GPA	1.3185	0.0684	19.27	<0.001	1.1844	1.4526
Years enrolled in college	−0.6983	0.4583	−1.52	0.128	−1.5966	0.2001
Course repetitions	0.0010	0.0203	0.05	0.960	−0.0388	0.0408
Number of online courses	0.0156	0.0120	1.30	0.193	−0.0079	0.0390
Number students in course	−0.0002	0.0003	−0.92	0.358	−0.0007	0.0003

**Table 17:** Four-way fixed effects linear regression analysis; student, course, instructor, and year fixed effects; low high school student performance sample; *N* = 14,105.

	Coef.	Robust S.E.	t	p	95% CI	
Intercept	−4.2781	1.1226	−3.81	<0.001	−6.4790	−2.0772
Online course	−0.0512	0.0943	−0.54	0.587	−0.2360	0.1336
Current college GPA	1.3710	0.1192	11.50	<0.001	1.1373	1.6047
Years enrolled in college	0.7204	0.5723	1.26	0.208	−0.4016	1.8424
Course repetitions	0.0813	0.0250	3.25	0.001	0.0323	0.1303
Number of online courses	−0.0013	0.0185	−0.07	0.944	−0.0377	0.0351
Number students in course	−0.0003	0.0004	−0.78	0.438	−0.0011	0.0005

**Table 18:** Four-way fixed effects linear regression analysis; student, course, instructor, and year fixed effects; medium high school student performance sample; *N* = 28,948.

	Coef.	Robust S.E.	t	p	95% CI	
Intercept	−1.0214	0.3339	−3.06	0.002	−1.6760	−0.3668
Online course	−0.1072	0.0701	−1.53	0.127	−0.2446	0.0303
Current college GPA	1.3269	0.0642	20.66	<0.001	1.2011	1.4528
Years enrolled in college	−1.2393	0.3831	−3.23	0.001	−1.9903	−0.4884
Course repetitions	0.0561	0.0184	3.04	0.002	0.0199	0.0922
Number of online courses	0.0073	0.0123	0.60	0.551	−0.0168	0.0314
Number students in course	0.0004	0.0003	1.35	0.176	−0.0002	0.0009

**Table 19:** Four-way fixed effects linear regression analysis; student, course, instructor, and year fixed effects; high school student performance sample; *N* = 18,348.

	Coef.	Robust S.E.	t	p	95% CI	
Intercept	−2.1836	0.4223	−5.17	<0.001	−3.0114	−1.3558
Online course	−0.2254	0.0790	−2.86	0.004	−0.3802	−0.0706
Current college GPA	1.5488	0.0864	17.93	<0.001	1.3795	1.7181
Years enrolled in college	−0.0375	0.0217	−1.73	0.083	−0.0800	0.0049
Course repetitions	−0.0303	0.0258	−1.18	0.239	−0.0809	0.0202
Number of online courses	−0.0110	0.0149	−0.74	0.459	−0.0401	0.0181
Number students in course	−0.0005	0.0003	−1.72	0.085	−0.0012	0.0001

## 2 Experimental design, materials and methods

### 2.1 Details of institutional data

Students enrolled in undergraduate courses at in the 2014 to 2017 summer terms were included in this data. This data includes enrollments from degree-seeking undergraduate students in lecture courses that were graded on a letter-grade scale (in contrast to a pass/no-pass distinction) and awarded at least four credit points. Data was provided by the Teaching and Learning Research Center and was compiled from a variety of sources on campus including: Office of Institutional Research, Office of the Registrar, Office of Undergraduate Admissions, Office of Institutional Technology, and the Office of Financial Aid and Scholarships. This project was supported by the UC Irvine Institutional Review Board. The data consisted of student-level demographic, performance, and college career information, as well as course-level information.

Student-level demographic information included gender (i.e., female, male), racial/ethnic background (i.e., White; Asian or Asian American; Black or African American; Latino or Hispanic; American Indian, Alaska Native, or Pacific Islander), first-generation college student status (i.e., neither parent holds a Bachelor's degree or higher), low-income status (i.e., derived from family household income and household size using 185% of the U.S. poverty line), English language learner status (i.e., Language other than English is students' first language), and whether or not the student is a California resident. Student performance indicators include, standardized admission test score (i.e., measured through American College Testing (ACT) and the Scholastic Aptitude Test (SAT) scores) and current college grade point averages. College career characteristics included students' years of enrollment in the institution, transfer student status, the number of online courses taken in college, and whether students repeated courses.

Course-level data includes course grades, course code, department, year and term the course was offered, the number of students enrolled in a course, course modality (i.e., online or face-to-face course modality), and unique instructor identification information.

Table 1 describes the overall data of all summer course enrollments prior to any list-wise deletion.

### 2.2 Data on multi-level logistic regression analysis

The following tables describe descriptive information of (a) all course enrollments, (b) all face-to-face course enrollments, and (c) all online course enrollments. This information is provided for both the full sample (all course enrollments) and a restricted sample that only includes courses that were offered as both online and face-to-face courses in the same term (Table 2, Table 3). These descriptive information are provided after listwise deletion. In addition, Table 4, Table 5, Table 6, Table 7 provide additional information on the two-level logistic regression models [2]. The models were conducted applying the *meqrlogit* syntax in Stata 15 [3]. In particular, course enrollments (level 1) were nested into courses (level 2).

### 2.3 Data on multi-way fixed effects modelling

The following table describes descriptive information for (a) all course enrollments, (b) all course enrollments of low-income students, (c) all course enrollments of first-generation college students, and (d) all course enrollments of students in the lower high school performance subgroup. This information is displayed separately by online and face-to-face course enrollments (Table 8). The descriptive information of time-variant variables is provided after listwise deletion (also accounting for missingness in the grouping variables (i.e., low-income status, first-generation college student status, high school performance indicator). In addition, Table 9, Table 10, Table 11, Table 12, Table 13, Table 14, Table 15, Table 16, Table 17, Table 18, Table 19 provide additional information on the multi-way fixed effects linear regression models [4,5]. The models sequentially introduced student, course, year, and instructor fixed effects and were conducted applying the *xtreg* syntax in Stata 15 [6]. Please note that these tables do not display coefficients for the course, instructor, and year series of dummy variables.
